# Radiation safety compliance awareness among healthcare workers exposed to ionizing radiation

**DOI:** 10.1186/s12912-024-01858-4

**Published:** 2024-03-27

**Authors:** Shaimaa Mohamed Elghareeb Allam, Mohamed Mustafa Abd Algany, Yasmin Ibrahim Abdelkader Khider

**Affiliations:** 1 Medical-Surgical Nursing Department, Faculty of Nursing, Mansoura University, Mansoura, Egypt; 2 Community Health Nursing Department, Faculty of Nursing, Mansoura University, Mnssoura, Egypt

**Keywords:** Radiation safety compliance awareness, Healthcare workers, Ionizing radiation

## Abstract

**Background:**

In recent years, there has been a marked growth in the use of ionizing radiation in medical imaging for both diagnosis and therapy, which in turn has led to increased radiation exposure among healthcare workers.

**Aim:**

The purpose of this study was to assess the level of safety compliance awareness among healthcare workers exposed to ionizing radiation.

**Research design:**

A descriptive cross-sectional design was used for this investigation.

**Setting:**

This study was conducted online, using social media sites such as WhatsApp, Instagram, and Facebook.

**Subjects:**

A purposive sample of 384 Egyptian healthcare workers was enrolled in the current study.

**Tool:**

A safety compliance awareness questionnaire was used in this study to collect pertinent data.

**Results:**

The result of this study showed that 65.4% and 64.1% of the studied sample chose the correct answers that mammography and CT scans involve the use of x-rays. However, 64.3% and 67.2% of the studied sample chose the wrong answers, saying that MRI and Ultrasound involve the use of X-rays. Moreover, 47.14%, 43.5%, and 57% of the studied sample never used a dosimeter, did not follow dosimeter controls, and did not wear a lead collar.

**Conclusion:**

Most of the healthcare workers studied had poor knowledge about radiation exposure safety. Moreover, most of the healthcare workers in the current study demonstrated inadequate practice compliance concerning radiation protection procedures.

**Recommendation:**

Should encourage hospital training programs to include radiation safety topics in their training plans for healthcare workers.

## Background

The application of ionizing radiation is essential in disease diagnosis and management. Recently, the healthcare setting has increased the usage of computed tomography and X-ray scans [1]. Every year, over 3.6 billion X-ray exams, thirty-seven million nuclear medications, and seven and a half million radiation treatments are done all over the world [2].

Radiation poses a health risk in both the workplace and the general environment. Radiation exposure in medical settings affects 20% of the global population, and this number will keep rising. In particular, compared to patients and other groups, the cancer rate among HCWs exposed to radiation is almost 40% higher [3]. Radiation exposure can cause health risks that manifest right away or later [4].

Chronic exposure can have negative health impacts on every system in the body, including prenatal malformations, cancer, benign tumors, and genetic disorders. Radiation sickness (bleeding, anemia, loss of bodily fluids, and bacterial infection) may be one of the more severe abnormalities [5]. For all HCWs who are exposed to radiation, safety knowledge is essential. Adhering to safety regulations may aid in lowering the frequency of health-related hazard sequences.

Healthcare Workers (HCWs) come into regular contact with various medical procedures involving radiation for diagnosis and treatment. Around 2.3 million HCWs are working with radiation worldwide. As a result, 50% of all healthcare workers are exposed to artificial and ionized radiation [6]. This precise aim sets the direction for the study, ensuring a focused investigation into the critical aspect of safety compliance in the context of radiation exposure in healthcare settings. The World Health Organization acknowledges that excessive ionizing radiation exposure raises the likelihood of adverse consequences. Ionizing radiation’s biological effects can be categorized as deterministic or stochastic [7].

Deterministic effects, also known as non-stochastic effects or tissue reactions, are the initial changes or damage in tissues or organs caused by high doses of radiation. They are directly related to the dose received and have a threshold dose. Stochastic effects, or probabilistic effects, are associated with exposure to ionizing radiation and can occur at any dose, but their probability increases with higher doses [5].

The International Commission on Radiological Protection has established dose limits for occupational exposure. The recommended limits are 20 millisieverts (mSv) per year, averaged over five years. However, the annual occupational exposure limit should not exceed fifty mSv. The Nuclear Regulatory Commission of the United States likewise recommended that cumulative fetal exposure throughout pregnancy be less than five mSv [8].

The purpose of the previous recommendations was to eliminate deterministic effects and maintain acceptable stochastic effects [9]. Going over the recommended limit could have harmful effects based on the duration, use of Personal Protective Equipment (PPE), and radiation dose. Moreover, HCWs can reduce radiation exposure through the careful use of radiation and the use of emerging technologies [10].

Radiation exposure is being reduced by new technology such as frameless image navigation and guidance systems [11]. Also, there is a growing focus on using PPE and dose-measuring methods. During all procedures, HCWs should wear standard radiation PPE like thyroid and eye shields, skirts, and lead aprons [12].

To protect their health, healthcare workers should be thoroughly informed about the dangers and precautions related to radiation exposure. Additionally, HCWs should adhere to radiation safety precautions [13] and be knowledgeable about radiation risks and safety measures through educational interventions on safety compliance [14]. Lack of radiation safety knowledge exposes HCWs and future generations to harmful effects [15].

Several research articles and reports have examined the effectiveness of educational programs in improving radiation safety practices among HCWs. The studies have demonstrated positive outcomes, such as increased knowledge, improved safety compliance, and reduced radiation exposure incidents. Some studies have also highlighted the long-term benefits of regular and ongoing education in maintaining safety compliance [3].

By incorporating these specific educational strategies and programs, healthcare organizations can empower HCWs with the necessary knowledge and skills to ensure radiation safety compliance. Additionally, citing studies that have shown the impact of such interventions on safety compliance strengthens the rationale for implementing these educational strategies and emphasizes their potential benefits in improving overall safety practices among HCWs [16].

Therefore, it’s critical to assess the level of safety compliance awareness among Egyptian HCWs exposed to ionizing radiation.

## Methods

### Aim of the study

The study aims to assess the level of safety compliance awareness among healthcare workers exposed to ionizing radiation.

### Research questions

Q1. What is the level of safety compliance awareness among HCWs who are exposed to ionizing radiation?

Q2. Is there a correlation between healthcare workers’ safety compliance and their awareness?

Q3. Is there a relationship between healthcare workers’ sociodemographic data and safety compliance awareness?

### Operational definitions

#### Radiation safety compliance awareness

In this study, radiation safety compliance awareness means having knowledge and understanding about different types of ionizing radiation, what they can do to you, and the dangers they pose. It also means following safety measures to avoid being exposed to radiation.

#### Ionizing radiation

Ionizing radiation is a sort of energy that can remove electrons from atoms and molecules in living tissues water and, air. It is a type of energy that cannot be seen and can go through different materials [17]. In this study, ionizing radiation refers to all medical devices that use this form of energy, such as MRI, X-ray, and CT.

### Research design

This study utilized a descriptive cross-sectional design which explains things or how things are related to each other at a specific time [18]. It was suitable for assessing the level of radiation safety compliance awareness among HCWs exposed to ionizing radiation.

### Setting

This study was conducted online, using social media sites such as WhatsApp, Instagram, and Facebook.

### Sample participation

A purposive sample of 384 Egyptian HCWs (technicians, nurses, physicians, physicists, and workers) was enrolled in the present study. The present study included HCWs who met the following inclusion criteria: they were adults from 20 to 60 years old, were from both genders, worked in medical departments, surgical departments, operating rooms, diagnostic radiology departments, Clinical Oncology and Nuclear Medicine departments, nuclear medicine units, outpatient clinics, and public health centers, were occupationally exposed to ionizing radiation, and accepted to take part in the current research. HCWs gathered through Facebook, Instagram, and WhatsApp groups in 2023. The online Google form spreadsheet was open began in May 2023 and ended in August 2023, at which point it was closed.

Based on the Central Agency for Public Mobilization and Statistics (CAPMAS), the current number of HCWs in Egypt is 375,000 [19]. However, the study only included individuals who had been exposed to radiation. According to the Thompson equation, a sample size of 384 participants was adequate for this investigation with a 95% confidence level, 0.05 error proportions, and 50% probability [20].

### Tools

One tool was used in this study for collecting pertinent data.

The data collected from participants likely focused on their level of awareness, knowledge, attitudes, and practices related to radiation safety in healthcare settings. The questionnaire used in the study would have included relevant questions to gather this information.

### Safety compliance awareness questionnaire

The researchers created this tool after studying information from different countries and sources [13, 21]. This tool included 25 statements in total, separated into four parts:

Part A contained five statements regarding HCWs’ sociodemographic characteristics, including sex, residence, age, marital status, and educational level.

Part B incorporated four statements covering HCWs’ specialty, the usage of the ionizing radiation machines as types of radiological exposure, years of experience, and whether the participants had already received radiation safety training at the hospital where they work.

Part C involved 10 statements about radiation safety knowledge. This part was answered with yes (one), and no (zero), with a total score of 10 grades classified as the following: the sum of each item’s scores turned into a percent score. According to the study of Ahmed et al. (2021), the percent score classified knowledge into poor, moderate, and good. Poor knowledge is less than 50%, moderate knowledge is 50–70%, and good knowledge is greater than 70% [4].

Part D consisted of six statements concerning compliance with safety procedures based on the participant’s experience in the past month. The scoring system of Part D was classified as the following: the participant rated his compliance with safety procedures using a three-point Likert scale (one = Never, two = Sometime, and three = Always), with a total score of 18 grades. Each item’s score was added up and turned into a percent score and classified as the following; adequate practice if the score ≥ 70%, or inadequate practice if the score < 70% [22].

### Validity and reliability

The tool was translated into Arabic and back-translated to English. A group of five experts with expertise in medical-surgical nursing, community health nursing, and medical biostatistics evaluated the tool for face validity. Face validity refers to the extent to which the tool appears to measure what it intends to measure. The experts likely reviewed the tool in terms of its clarity, relevance, comprehensiveness, simplicity, and ease of use. Based on their expertise, they provided feedback and made changes to improve the tool. Following the expert evaluation, the researchers incorporated the suggested changes and modifications to enhance the clarity, relevance, comprehensiveness, simplicity, and user-friendliness of the tool. As an example of tool modification, questions like “use a dosimeter and wear a leaded collar” were added to the study tool. Another modification is replacing the answer to Part D of the study tool: “Yes, or No” with a three-point Likert scale (one = never, two = sometimes, and three = always). These modifications aimed to address any identified issues and improve the overall quality of the tool.

To assess the reliability of the study tool, Cronbach’s alpha was used. Cronbach’s alpha is a statistical measure that assesses the internal consistency or reliability of a scale or questionnaire. It ranges from 0 to 1, with higher values indicating greater reliability. In this case, the scores obtained for Part C of the questionnaire were 0.83, indicating a good level of reliability. Similarly, Part D received a score of 0.87, suggesting a high level of reliability.

### Pilot study

At first, a small study was done with 39 (10%) of the intended HCWs. However, they were later removed from the study. The reason we conducted this initial investigation was to check if the research tool was clear, doable, and useful, and to estimate how much time it would take to finish and submit it. Based on the initial investigation, we made the necessary changes and improvements before gathering data.

### Data collection

The researchers created a study tool called the safety compliance awareness questionnaire. This tool was made based on a recent relevant literature review. After that, the researchers translated the study tool into Arabic for the first time. Next, an English teacher with experience in medical terms back-translated the Arabic version of the study tool to English. The teacher did not know the original text. Later, a group of experts checked the Arabic version to make sure the translations matched the original scale. A group of five professionals in the same subjects reviewed the study tool to ensure its accuracy and relevance. Any needed changes were made based on their feedback. The researchers tested how reliable the tools were by using a statistical test called Cronbach’s alpha coefficient. A pilot study was done with 39 HCWs (which is 10% of the total participants) to see if the study tool could be used effectively. They were excluded from this study. The changes were made as needed. After getting permission to start the study, the researchers made a questionnaire using Google Forms to collect the required information. The researchers shared the link with HCW’s groups on Facebook, WhatsApp, and Instagram (https://forms.gle/VrMpKUX1z2SkF6gm8). The researchers gave a short statement explaining the objective and nature of the study at the start of the online survey. Moreover, the researchers emphasized the importance of keeping the privacy and confidentiality of participants’ information. Where data collected from each participant is stored within a Google Form file and then transferred by the researcher solely into an Excel file without disclosing or sharing the information with any participants, it can be seen as a step taken to preserve the confidentiality of the data. This method ensures that participant data remains protected and is not accessible to others. If the HCWs agreed to be part of this study, they were asked to say “yes” and then move on to the next part of the study. If HCWs did not want to take part in the study, they clicked “no” and were removed from the questionnaire. The participant needed around 10 to 15 min to complete the online survey. The process of collecting data began in May 2023 and ended in August 2023.

### Data analysis

The researchers used their computers to download the data collected from Google Forms. The information was put into Excel and SPSS Version 20.0 released in 2013, Armonk, NY: IBM Corp, which is software for analyzing numbers and statistics in social science. The researchers looked at and organized the collected information and then assigned it a code. The researchers used numbers and percentages to describe different categories, and one-way ANOVA is a way to compare the means of two or more independent groups. If the *p*-value is 0.05 or higher, it is seen as statistically significant, and if the *p*-value is 0.01 or lower, it is considered highly significant. Pearson correlation was used to measure the strength of the linear relationship between two variables.

## Results

**Fig. 1:**
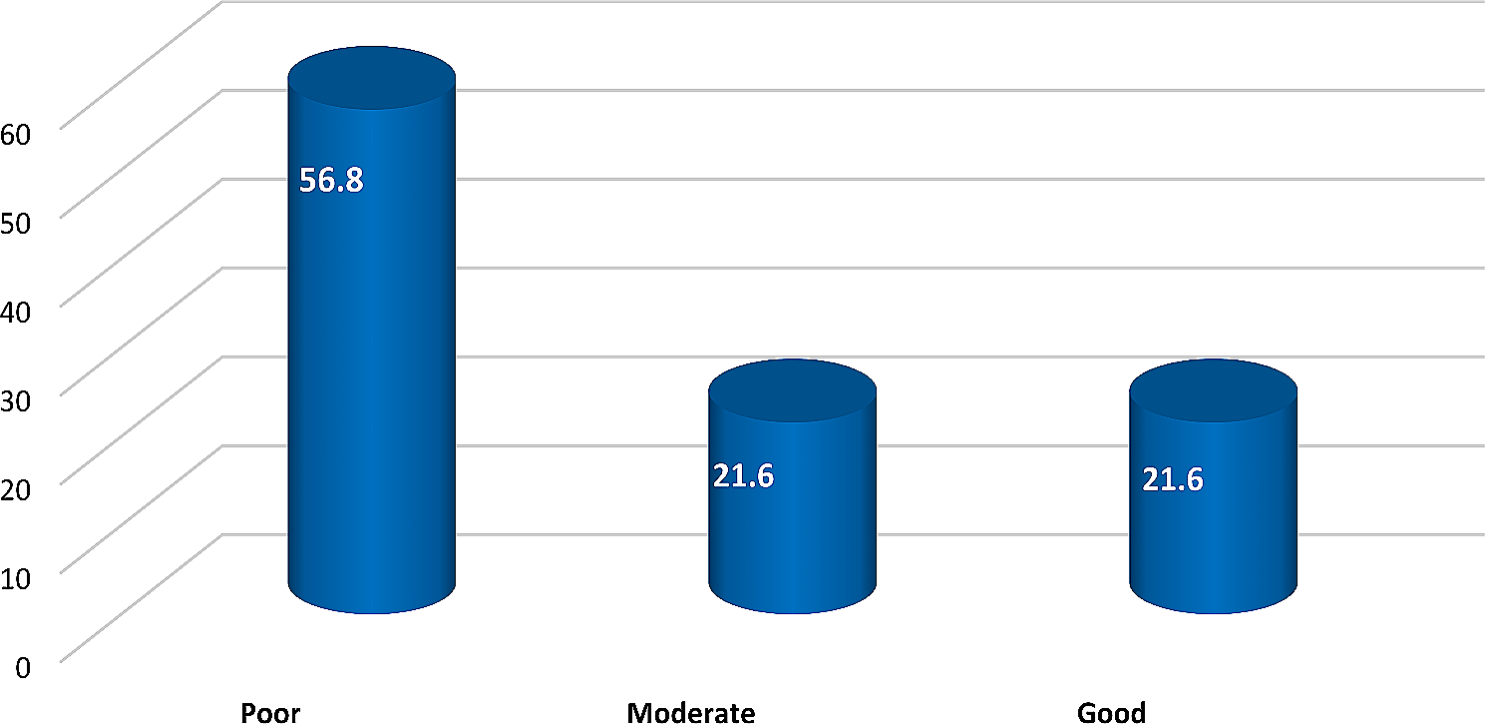
Total knowledge score categories of the participants (*N* = 384)

**Fig. 2:**
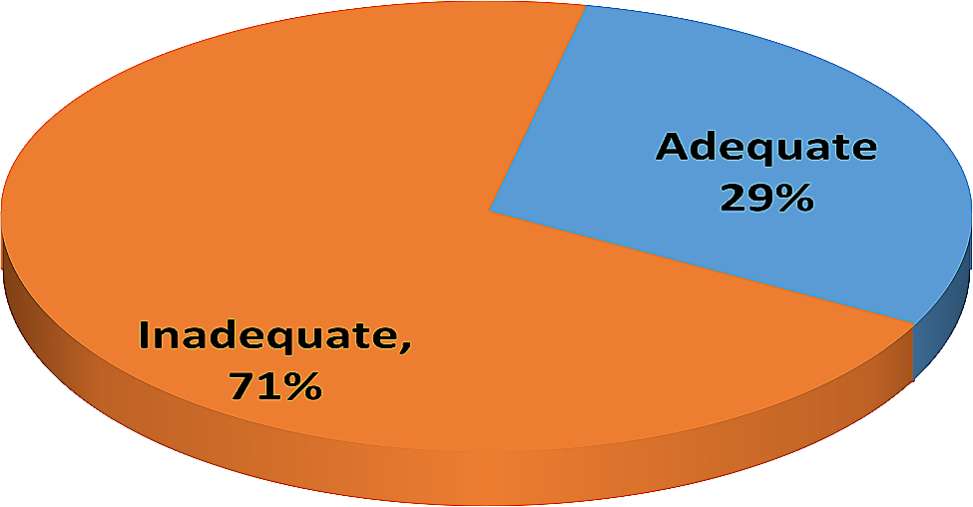
Total safety practices compliance score of the studied sample (*N* = 384)

This study involved a total of 384 Egyptian HCWs who accepted and enrolled in the present study, Table 1. shows that 64.1%, 60.4%, 57.81%, and 64.8% of the studied sample were aged from 20 to 30 years, female, single, and lived in rural areas, respectively, while 50.52% had a bachelor’s degree and 75% were nurses. Table 2. shows that 49.73% of the participants were exposed to X-rays, 39.3% of them experienced less than three years of dealing with radiation, and 58.9% of them never had any training courses in radiation. Table 3. shows that 65.4% and 64.1% of the studied sample chose the correct answers that mammography and CT scans involve the usage of x-rays, and 52.1% of them chose stepping out of the room if fluoroscopy is on and if he or she is not operating or assisting in the procedure. Moreover, 70.1% and 60.4% of the studied HCWs marked the correct answers that skin abnormalities and bone marrow depression are radiation risks, respectively. However, 64.3% and 67.2% of the studied HCWs chose the wrong answers, saying that MRI and Ultrasound involve the use of X-rays. About 53.6%, 58.9%, and 74.7% of the participants in the study did not know the normal minimum safe distance from the X-ray machine while doing portable X-rays, the highest permissible amount of occupational radiation exposure, or that pregnant nurses cannot work in fluoroscopy in the first trimester. Figure 1 reveals that 56.8% of the studied sample had poor knowledge about radiation exposure safety awareness. Table 4 exposes that 47.14%, 43.5%, 36.2%, and 57% of the studied sample never used a dosimeter, did not follow dosimeter controls, did not wear a leaded apron, or did not wear a leaded collar. Figure 2 shows that 71% of the studied sample had inadequate compliance practices with radiation safety procedures. Table 5. explains that there is a strong relationship between the studied sample’s age and their total compliance with radiation safety measures (*P* = 0.000**). Also, there is a strong relationship between the participants’ educational level, their total knowledge score about radiation exposure safety awareness, and their total practice compliance score (*P* = 0.000**). Finally, there is a strong relationship between the specialties of the participants and their total knowledge score about radiation exposure safety (*P* = 0.000**). Table 6. reveals a high association between the years of experience of the study sample and their total practice compliance score for radiation safety measures (*P* = 0.000**). Also, there is a strong relationship between the participants’’ radiation training and their total knowledge score about radiation-exposure safety awareness and their total practice compliance score (*P* = 0.000**). Table 7. shows that there was a significant positive correlation between the studied sample’s total knowledge score and their total practice compliance score.

**Table 1:** Socio-demographic data distribution (*N* 384)

Items	N	%
**Age**		
20–30	246	64.1
>30–40	89	23.1
>40–50	38	9.9
>50–60	11	2.9
**Gender**		
Male	152	39.6
Female	232	60.4
**Marital status**		
Married	146	38.02
Single	222	57.81
Divorced	12	3.13
Widowed	4	1.04
**Residence**		
Urban	135	35.2
Rural	249	64.8
**Educational level**		
Basic education	27	7.03
Secondary	14	3.65
Institution	106	27.6
Bachelor	194	50.52
Master	23	6
Doctoral	20	5.2
**Specialty**		
Cardiologists	4	1
Radiologist	16	4.2
Orthopedics	8	2.1
Urologists	8	2.1
General Surgeons	3	0.8
Radiographers	57	14.8
Nurses	288	75

**Table 2:** Radiation exposure distribution (*N* 384)

Items	N	%
**Radiation type**		
MRI	12	3.13
CT	43	11.2
X-RAY	191	49.73
Mixed types	138	35.94
**Years of experience**		
>3yrs	151	39.3
3->6yrs	103	26.8
6 and more	130	33.9
**Radiation training**		
Never	226	58.9
Once	86	22.4
Twice and more but irregular	52	13.5
More than once regular	20	5.2

**Table 3:** Distribution of the studied sample according to their knowledge about radiation exposure safety awareness (*N* = 384)

Items	Yes		No	
	N	%	N	%
Mammography involves the usage of X-rays	251	65.4	133	34.6
CT scan involves the usage of X-rays	246	64.1	138	35.9
MRI does not involve the usage of X-rays	137	35.7	247	64.3
Ultrasound does not involve the usage of X-rays	126	32.8	258	67.2
I know the standard minimum safe distance from the X-ray machine while performing portable X-rays	178	46.4	206	53.6
I know the highest permitted level of occupational radiation dose	158	41.1	226	58.9
If fluoroscopy is on, and if you are not operating or assisting in the procedure, do you step out of the room?	200	52.1	184	47.9
A pregnant nurse cannot work in fluoroscopy in the first trimester	97	25.3	287	74.7
Skin injuries like erythema, skin pigmentation, hair loss, and desquamation are radiation risks that you are exposed to at the workplace	269	70.1	115	29.9
Bone marrow depression is radiation risks that you are exposed to at the workplace	232	60.4	152	39.6

**Table 4:** Distribution of the studied sample according to their compliance with safety procedures (*N* = 384)

Items	Always		Sometimes		Never	
	N	%	N	%	N	%
Use a dosimeter	98	25.52	105	27.34	181	47.14
Follow dosimeter controls	112	29.2	105	27.3	167	43.5
Wear a leaded apron	122	31.8	123	32	139	36.2
Wear a leaded collar	74	19.3	91	23.7	219	57
Stand behind a control booth or step outside the room	152	39.6	109	28.4	123	32
Follow safety measures for portable X-rays	137	35.7	111	28.9	136	35.4

**Table 5:** Relationship between socio-demographic features of the examined sample, total knowledge score, and practice compliance (*N* = 384)

Socio-demographic features	Total knowledge score	Total practices compliance
**Age**		
20–30	4.78 (3.14)	6.39 (4.24)
>30–40	5.08 (3.52)	3.09 (3.18)
>40–50	5.58 (2.48)	3.89 (3.62)
>50–60	4.91 (3.36)	3.36 (4.08)
Significance	F = 0.772	F = 18.05
	*P* = 0.51	*P* = 0.000**
**Gender**		
Male	4.84 (2.77)	5.05 (4.02)
Female	4.99 (3.43)	5.46 (4.34)
Significance	F = 0.202	F = 0.845
	*P* = 0.653	*P* = 0.358
**Educational level**		
Basic education	3.67 (3.68)	6.33 (4.76)
Secondary	3.29 (3.05)	3.14 (3.06)
Institution	4.63 (2.74)	4.35 (3.99)
Bachelor	4.92 (3.02)	6.29 (4.24)
Master	7.09 (3.64)	3.48 (3.09)
Doctoral	7.00 (3.83)	2.8 (2.69)
Significance	F = 5.96	F = 7.12
	*P* = 0.000**	*P* = 0.000**
**Specialty**		
Cardiologists	9.00 (2.00)	6.75 (6.19)
Radiologist	8.81 (1.72)	6.00 (2.61)
Orthopedics	5.25 (3.37)	3.13 (2.03)
Urologists	5.88 (3.52)	3.00 (4.18)
General Surgeons	10.00 (0.00)	0.33 (0.58)
Radiographers	5.18 (2.33)	5.26 (3.43)
Nurses	4.52 (3.18)	5.42 (4.42)
Significance	F = 8.12	F = 1.657
	*P* = 0.000**	*P* = 0.131

**Table 6:** Relationship between the radiation exposure of the studied sample and their total knowledge score and their practice compliance score (*N* = 384)

Radiation exposure	Total knowledge score	The total practices compliance score
**Years of experience**		
Less than 3 years	4.64 (3.09)	7.03 (4,26)
3– less than 6	4.94 (2.96)	4.82 (3.95)
6 years and more	5.26 (3.43)	3.67 (3.59)
Significance	F = 1.327	F = 26.13
	*P* = 0.267	*P* = 0.000**
**Radiation training**		
Never	3.86 (3.02)	4.62 (4.17)
Once	5.89 (2.52)	5.88 (4.26)
Twice and more but irregular	7.23 (2.72)	6.87 (3.98)
More than once regular	6.9 (3.34)	6.4 (3.57)
Significance	F = 27.467	F = 5.582
	*P* = 0.000**	*P* = 0.001**

**Table 7:** Correlation between independent variables Knowledge and Practice Compliance (N = 384)

Predictor	Knowledge score	
	r	*P* value
Practices compliance score	0.248	0.000**

r: for Pearson correlation. *P* value significant if ≤ 0.05

If r ≤ 0.5 = weak correlation. If r ˃ 0.5 = strong correlation

## Discussion

Recent technological advancements have led to a heightened utilization of radiation-intensive medical applications, consequently resulting in increased exposure to radiation for both patients and healthcare professionals. Consequently resulting in increased exposure to radiation for both patients and healthcare professionals [23]. Thus the major goal of this study was to measure the level of safety awareness among HCWs who were exposed to ionizing radiation. The results of the current study indicated that the HCWs who participated in this study and were occupationally exposed to ionizing radiation sources lacked radiation protective expertise.

According to the current study’s findings, around two-thirds of the subjects evaluated were between the ages of 20 and 30 years. By taking into consideration using digital forms (online surveys) as a way of collecting data, this may lead to an increase in response from a young age. Digital surveys can increase young responses due to their early technological skills and openness to experimentation. With familiarity with technology and radiation-related equipment, they are more proficient in using technology-related tools. However, individual experiences and comfort levels can vary, and proficiency can be achieved through learning and adaptation. This result is consistent with Erkan, et al. (2019), who examined the awareness of radiation safety of HCWs in an education and research hospital and showed that almost half of their participants were between 18 and 30 years old [13]. Also, this result was the same line as Ahmed, et al., (2021), who assessed nurses’ and technicians’ knowledge and behaviors regarding radiation dangers and safety procedures at Main Assuit University hospitals and showed that almost two-fifths of their sample were less than 30 years old [4]. On the contrary, Hussein et al., (2022), who assessed the dangers of ionizing radiation in the radiological health team, revealed that over two-fifths of their participants were between 30 and 40 years old [22].

In terms of occupational category, three-quarters of the current sample examined were nurses. This result can be interpreted to mean that, according to CAPMAS (2023), the Egyptian public sector workforce is estimated at 73,400 doctors compared to 148,600 nurses [24]. From the researchers’ point of view, based on the previous statistic, the number of nurses in Egyptian hospitals is more prominent than that of other HCWs, and this may be the reason why three-quarters of the current sample are nurses. Nursing staff should be educated on radiation safety through comprehensive programs, collaborations, and practical demonstrations. Regular refresher courses, newsletters, and online resources are essential. Administrative processes should ensure compliance with protocols. Fostering a safety culture within healthcare organizations promotes open communication and continuous improvement This finding is concurrent with the study by Antunes-Raposo, et al., (2020), who evaluated the use of PPE among HCWs exposed to ionizing radiation and found that almost a third of them were nurses [25]. In contrast, an Egyptian study by Soliman, et al., (2019) aimed to describe HCWs’ knowledge and practices regarding safety and exposure to ionizing radiation and found that over than half of their study participants were physicians [26].

Concerning years of experience, in the present study, over two-fifths of the analyzed sample had less than three years of experience. From the researchers’ point of view, this could potentially affect their knowledge and awareness levels regarding radiation safety procedures. Influence on adherence to safety Procedures: Nurses with less experience might be less familiar with the intricacies of radiation safety protocols or may have had fewer opportunities to practice and reinforce their adherence to these procedures. This could potentially impact their compliance with safety measures. This result comes close to that of Alyousef, et al., (2023), who evaluated HCWs’ awareness of radiation safety and typical radiation dose and revealed that more than half of their sample had less than one year of work experience [27]. On the contrary, the results of Soliman et al., (2019) showed that more than two-fifths of their participants had more than 10 years of professional experience [26].

More than half of the participants in the current study had never attended any radiation training courses. We attribute this result to the fact that, although there is a training center in the hospital, radiation safety topics are not on their priority list. This lack of formal training can result in a limited understanding of radiation hazards, safety protocols, and the importance of adherence to safety measures. As a consequence, HCWs, including nurses, may not possess the necessary knowledge to effectively protect themselves, patients, and others from potential radiation risks. In agreement with the present finding comes the result of Ahmed et al., (2021), as the majority of their sample did not attend any training course [4]. Contradicting our finding is the study by Zekiolu and Parlar (2021), who examined the level of radiation safety knowledge among HCWs employed in a radiation environment and found that almost two-thirds of their participants had regularly attended radiation safety training courses in the hospital in which they work [28].

Considering the total knowledge score, this study discovered that more than half of the studied sample had poor knowledge about radiation exposure safety, and we justify the reason for the result that HCWs did not attend any training courses about radiation safety. In the same vein, the finding of Ahmed et al., (2021), demonstrated that most of their sample’s overall radiation knowledge was poor [4]. On another occasion, the finding of Bolbol et al., (2021) expressed that the majority of their HCWs had adequate radiation knowledge [9]. In addition, Hussein et al., (2022), to the contrary, found that more than half of their HCWs had satisfactory knowledge of ionizing radiation [22].

Regarding compliance with safety procedures, the highest percentage of the present sample reported that they never followed radiation safety precautions in their workplaces, except while standing behind a control booth or exiting the room. This result may be a result of a lack of HCWs’ radiation safety awareness. Also, increases HCWs’ work overload, which makes them unable to follow radiation safety measures. In addition to the unavailability or insufficient number of safety equipment. Moreover, some of the protective equipment, such as the loaded apron, is too heavy, which obstructs compliance with safety procedures. According to Salem (2022), a full-body apron weighs roughly 7 kg and can cause back problems, according to who conducted a questionnaire survey on radiation protection among medical workers in the cardiac catheter laboratory [29].

This result agrees with the findings of Soliman et al., (2019), who clarified that their HCWs’ practices regarding wearing a personal monitoring bandage and lead apron were inadequate [26]. Our results partially disagree with a Turkish study by Zekiolu & Parlar (2021) who found that most samples regularly use a pocket dosimeter [28]. This result may be explained by the fact that the usage of a dosimeter in radiation regions is required, and the guidelines for doing so are outlined in Turkish legislation. However, a small percentage of participants reported wearing lead aprons, lead glasses, thyroid collars, or lead gloves.

In terms of overall practice compliance, more than two-thirds of the current study demonstrated inadequate practice compliance concerning radiation protection procedures. The researchers’ review suggests that this finding may be related to a lack of knowledge about radiation safety among HCWs. In addition, materials are not available in sufficient quantities for all those exposed to radiation, and there are no fixed rules in the workplace regulations that oblige medical staff to protect themselves.

In the same context as a study done by Ahmed et al., (2021), who found that more than two-thirds of their study sample had poor radiation protection practices [4]. In addition, Soliman and College (2019) noticed inadequacies in self-reported radiation safety practices among all participants in their study [26]. Furthermore, this discovery is consistent with Hussein et al., (2022), who discovered that nearly two-thirds of their participants had inadequate ionizing radiation protection practices [22].

On the other hand, this study is in contrast to that conducted to assess HCWs’ knowledge, attitudes, and practices regarding radiation safety and discovered that their sample had a positive attitude, average awareness, and average knowledge of radiation protection [3].

In terms of the relationship between socio-demographic variables and total knowledge score, the current study’s findings revealed that there was a highly statistically significant difference between the samples’ levels of education, their medical specialty, their previous radiation training, and their knowledge about radiation exposure safety awareness. This result may be because as HCWs’ levels of education increase, their ability to put the learned concepts into practice increases, and training courses also allow HCWs to acquire new knowledge and skills. If the training courses are effective, HCWs will apply this information in their workplace, a process known as learning transfer.

This result is consistent with the finding of Rahimi et al., (2021), who implied a significant association between a nurse’s age, work experience, medical radiation training, and radiation knowledge domain [30]. Another study reported that the age of HCWs was not significantly related to their knowledge of radiation [9]. Furthermore, our result was inconsistent with that of examining the level of awareness and knowledge about radiation safety among HCWs working in a radiation environment and discovered that there was no significant difference based on the amount of knowledge, experience, or hospital type [28].

There was also a significant variation in the ages of the present study samples, as well as their level of education, years of experience, previous radiation training, and adherence to radiation safety practices. The results of Soliman et al., (2019) partially agree with the present result, as they reported that the age of the participants is an important predictor of radiation practices [26]. In contrast, in Anatolian, there was no significant link identified between radiation protective measures and long-term experience, daily fluoroscopy exposure, or training on the present knowledge and attitudes of surgical staff in urology about ionizing radiation [10].

In addition, there was a correlation between the total knowledge score of the present study sample and their compliance with radiation practices. This finding has a logical explanation because increased knowledge and awareness of radiation risks may increase adherence to radiation safety compliance. This result is in the same line with the finding of Ahmed et al., (2021), who found that there was a strong positive correlation between the total score of their assessed sample knowledge and the total score of practices connected to radiation safety measures [4]. Similar to another finding, found that the knowledge value and practice value have a high positive correlation [26]. Additionally, Hussein et al., (2022) concluded that, based on their findings, radiation knowledge showed statistically significant positive correlations with radiation protection practices [22].

### Limitations of the study

A limitation of this research is that it was an online self-administered questionnaire-based study; therefore, HCWs may have answered some questions after checking for the correct answers. Another limitation of this study is the response rate. To reach the estimated sample size, the researchers had to frequently share the link to the online survey on electronic websites.

## Conclusions and recommendations

Based on the outcomes of this study, it is possible to conclude that the majority of the studied Egyptian HCWs had poor knowledge about radiation exposure safety. In terms of overall practice compliance, most of the HCWs in the current study demonstrated inadequate practice compliance concerning radiation protection procedures.

The following recommendations are based on the study’s findings; create an educational program to teach HCWs about radiation safety and how to use it in their work. Encourage hospitals to teach healthcare workers about radiation safety in their training programs. Every year, HCWs should take training courses to make sure they are up to date with recent changes and remember to practice radiation safety, which is sometimes forgotten. Do another study to find out the reasons for noncompliance with radiation safety rules.

## Data Availability

The datasets generated and/or analyzed during the current study are not publicly available due to protecting the confidentiality of the participants but are available from the corresponding author upon reasonable request.

## Abbreviations

CAPMAS: Central Agency for Public Mobilization and Statistics
CT: Computed Tomography
DNA: Deoxyribonucleic acid
HCWs: Healthcare Workers
MRI: Magnetic resonance imaging
mSv: Millisieverts
PPE: Personal Protective Equipment
